# Novel RAF‐directed approaches to overcome current clinical limits and block the RAS/RAF node

**DOI:** 10.1002/1878-0261.13605

**Published:** 2024-02-16

**Authors:** Rossella Scardaci, Ewa Berlinska, Pietro Scaparone, Sandra Vietti Michelina, Edoardo Garbo, Silvia Novello, David Santamaria, Chiara Ambrogio

**Affiliations:** ^1^ Department of Molecular Biotechnology and Health Sciences, Molecular Biotechnology Center University of Torino Italy; ^2^ Department of Oncology University of Torino, San Luigi Hospital Orbassano Italy; ^3^ Centro de Investigación del Cáncer CSIC‐Universidad de Salamanca Spain

**Keywords:** cancer resistance, inhibitors, novel therapies, paradoxical effect, RAF, RAS

## Abstract

Mutations in the RAS–RAF–MEK–ERK pathway are frequent alterations in cancer and RASopathies, and while RAS oncogene activation alone affects 19% of all patients and accounts for approximately 3.4 million new cases every year, less frequent alterations in the cascade's downstream effectors are also involved in cancer etiology. RAS proteins initiate the signaling cascade by promoting the dimerization of RAF kinases, which can act as oncoproteins as well: BRAF^V600E^ is the most common oncogenic driver, mutated in the 8% of all malignancies. Research in this field led to the development of drugs that target the BRAFV600‐like mutations (Class I), which are now utilized in clinics, but cause paradoxical activation of the pathway and resistance development. Furthermore, they are ineffective against non‐BRAFV600E malignancies that dimerize and could be either RTK/RAS independent or dependent (Class II and III, respectively), which are still lacking an effective treatment. This review discusses the recent advances in anti‐RAF therapies, including paradox breakers, dimer‐inhibitors, immunotherapies, and other novel approaches, critically evaluating their efficacy in overcoming the therapeutic limitations, and their putative role in blocking the RAS pathway.

## Graphical abstract

RAS proteins initiate the RAS–RAF–MEK–ERK signaling by promoting dimerization of RAF kinases, also active as oncoproteins. Drugs targeting BRAF^V600‐like^ mutations (Class I) cause the paradoxical effect and resistance, and are ineffective against other classes of BRAF malignancies. This review explores recent advances in anti‐RAF therapies, including paradox breakers, dimer‐inhibitors, and immunotherapies, assessing their efficacy in overcoming therapeutic limitations and potential roles in blocking the RAS pathway.

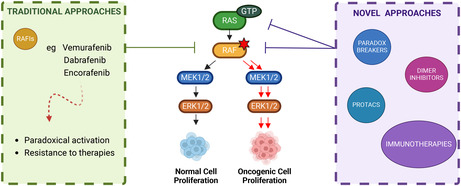

**Abbreviations**
ACT: adoptive cell transfer
AE: adverse events
AS: activation segment
ATP: adenosine triphosphate
CAR: chimeric antigen receptor
CIDI: αC‐IN/DFG‐IN conformation
CIDO: αC IN/DFG‐OUT conformation
CNS: central nervous system
CODI: αC‐OUT/DFG‐IN conformation
CRBN: cereblon E3 ligase
CRC: colorectal cancer
CRD: cysteine‐rich domain
CTLA‐4: cytotoxic T‐lymphocyte antigen 4
DIF: dimer interface
*D*_Max_
: maximal percentage of degradation
dRAFi: dimer‐selective RAF inhibitor
ERKi: ERK inhibitor
FDA: Food and Drugs Administration
GDP: guanosine diphosphate
GEM: genetically engineered mouse
GTP: guanosine triphosphate
HCC: hepatocellular carcinoma
HGG: high‐grade glioma
ICI: immune checkpoint inhibitor
KSR: kinase suppressor of RAS
MAPK: mitogen‐activated protein kinase
MDA: melanocyte differentiation antigens
MEKi: MEK inhibitor
mRAFi: monomer and dimer‐binding RAF inhibitor
MW: molecular weight
NSCLC: non‐small‐cell lung cancer
NtA: N‐terminal acidic motif
OS: overall survival
PB: paradox breakers
PD‐1: programmed cell death 1
PDAC: pancreatic ductal adenocarcinoma
PD‐L1: programmed cell death ligand 1
PDX: patient derived xenograft
PFS: progression‐free survival
POI: protein of interest
PP1c: catalytic subunit PP1
PP5: protein phosphatase 5
PPI: protein–protein interaction
PROTAC: proteolysis targeting chimera
RAFi: RAF inhibitor
RBD: RAS‐binding domain
RGS: rigosertib
RTK: receptor tyrosin kinase
TIL: tumor infiltrating lymphocyte
VEGF: vascular endothelial growth factor
VHL: Von Hipple‐Lindau E3 ligase
WT: wild type

## 1 Introduction

RAS family members are among the most studied proteins due to their roles in signal transduction, cell proliferation, migration, survival, and as oncogenes in human cancers. The RAS family consists of four GTPase isoforms, namely KRAS4a, KRAS4b, HRAS, and NRAS, which activate, among several pathways, the mitogen‐activated protein kinase (MAPK) RAF–ERK–MEK cascade [1]. RAS mutations occur in approximately 30% of all cancers [2], and, although there is a high degree of similarity between the four isoforms, KRAS is the most frequent tumor driver [3, 4]. At the molecular level, alterations in this gene family inhibit the enzymatic process that transforms active RAS‐GTP to inactive RAS‐GDP, leading to unbalanced protein activation [5]. In the last few years, RAS mutations have gained increasing importance as diagnostic and prognostic indicators and therapeutic determinants in human cancer. Some recent studies have contributed to the development of molecular targeted therapy, personalized medicine, or precision medicine, and have been extensively reviewed elsewhere [4, 6]. With the discovery of compounds that bind covalently and therefore, irreversibly to cysteine 12 of GDP‐KRAS^G12C^ mutants (off‐inhibitors), a new frontier for directly targeting KRAS has emerged after years of research. The two small molecules adagrasib (MRTX849) [7] and sotorasib (AMG510) [8] have been approved as the first targeted therapies for KRASG12C non‐small‐cell lung cancer (NSCLC), with modest efficacy in colon cancer. However, despite the therapeutic benefit shown in many patients, most of them eventually developed acquired resistance to single‐agent therapy, with mechanisms of resistance to KRAS^G12C^ inhibition still incompletely poorly understood [9, 10, 11, 12]. The issue of tailored therapy for tumors driven by different KRAS mutations or RAS isoforms remains unresolved [13, 14] despite the promising breakthroughs in covalent drugs.

The RAF enzymes, protein‐serine/threonine kinases, are the primary effectors that GTP‐bound RAS recruits to initiate the RAS‐dependent pathway activation [15]. Their activation requires direct contact with active RAS proteins localized at the membrane and leads to the initiation of a sequence of downstream phosphorylation events starting from MEK to different cellular targets that will specify a variety of biological responses [16]. Thus, they represent a second central node in the RAS–RAF–MEK–ERK pathway and play a pivotal role in tumors that rely heavily on this cascade. Also, RAF kinases themselves can act as oncoproteins: The most common oncogenic variant in the family is BRAF^V600E^, mutated in the 8% of all malignancies [2]. Over time, several attempts have been made to develop RAF inhibitors for cancer therapy. Treatment of BRAF mutant (V600E) melanomas with RAF inhibitors (such as vemurafenib and dabrafenib—Table 1) has been shown to be clinically beneficial; however, resistance and paradoxical activation (see Section 3) of the pathway still arise as a result of RAF dimerization and reactivation of ERK signaling [17, 18, 19]. In addition, they are ineffective against NON‐V600 BRAF mutations [20]. Therefore, there is an unmet need for further innovative approaches to target the RAS–RAF pathway more effectively and with a better outcome for patients.

**Table 1:** List of drugs discussed in the review and grouped according to the mechanism of action. Clinical trial references are included when possible.

Drug name	Class	Research phase	Cancer type	Reference
Sorafenib	ATP competitive inhibitor	Approved in clinic	Advanced renal cell carcinoma; angiosarcoma; gastrointestinal stromal tumor (GIST); leiomyosarcoma (LMS); unresectable hepatocellular carcinoma (HCC); progressive, locally recurrent radioactive iodine‐refractory differentiated thyroid carcinoma (DTC); progressive, metastatic radioactive iodine‐refractory differentiated thyroid carcinoma (DTC)	[84, 85]
Vemurafenib	ATP competitive inhibitor	Approved in clinic	Metastatic melanoma; refractory lung non‐small cell carcinoma; unresectable melanoma; refractory Erdheim–Chester disease	[88, 115]
Dabrafenib	ATP competitive inhibitor	Approved in clinic	Locally advanced anaplastic thyroid cancer; low‐grade glioma; melanoma; metastatic anaplastic thyroid cancer; metastatic melanoma; metastatic non‐small cell lung cancer; metastatic solid tumors; unresectable melanoma	[88, 115]
Encorafenib	ATP competitive inhibitor	NCT04655157 (Phase 1/2); NCT05270044 (Phase 3)	Metastatic colorectal cancer (CRC); metastatic melanoma; metastatic non‐small cell lung cancer; unresectable melanoma	[89]
LY3009120	PanRaf	NCT02014116 (Phase 1)	Melanoma; non‐small cell lung carcinoma; colorectal neoplasms; neoplasms metastasis; neoplasms	[128]
TAK‐580	PanRaf	NCT03429803 (Phase 1)	Melanoma	[129]
Belvarafenib	PanRaf	NCT02405065 (Phase 1); NCT03284502 (Phase 1); NCT03118817 (Phase 1); NCT04835805 (Phase 1)	Melanoma	[135]
Lifirafenib	PanRaf	NCT02610361 (Phase 1); NCT03905148 (Phase 1)	Solid tumors	[137]
LXH254	PanRaf	NCT02607813 (Phase 1); NCT04417621 (Phase 2); NCT02974725 (Phase 1); NCT04294160 (Phase 1)	Melanoma	[139]
Exarafenib	PanRaf	NCT04913285 (Phase 1); NCT04511013 (Phase 2)	Non‐small cell lung cancer; melanoma; solid tumors	[145]
PLX4072	Paradox breaker	Preclinical		[151]
PLX8394	Paradox breaker	NCT02012231 (Phase 1); NCT05503797 (Phase 2)	Melanoma; thyroid cancer; colorectal cancer; non‐small cell lung cancer	[151]
C1a	Paradox breaker	Preclinical		[125]
PHI1	Allosteric inhibitor	Preclinical		[162]
Braftide	Allosteric inhibitor	Preclinical		[164]
SJF‐0628	BRAF targeting PROTAC	Preclinical		[175]
P4B	BRAF targeting PROTAC	Preclinical		[176]
CRBR(BRAF)‐24	BRAF targeting PROTAC	Preclinical		[177]
Trametinib	MEK inhibitor	NCT04417621 (Phase 2); NCT02974725 (Phase 1); NCT04294160 (Phase 1)	Advanced non‐small cell lung cancer (NSCLC); locally advanced anaplastic thyroid cancer; low‐grade glioma; melanoma; metastatic anaplastic thyroid cancer; metastatic melanoma; metastatic non‐small cell lung cancer	
Cobimetinib	MEK inhibitor	NCT03284502 (phase 1); NCT04835805 (Phase 1)	Melanoma; locally advanced solid tumor; metastatic solid tumor	
Mirdametinib	MEK inhibitor	NCT03905148 (Phase 1)	Solid tumors	
Binimetinib	MEK inhibitor	NCT04655157 (phase 1/2); NCT05270044 (phase 3); NCT04511013 (Phase 2)	Melanoma	
LTT462	ERK inhibitor	NCT02974725 (Phase 1); NCT04417621 (Phase 2)	Non‐small cell lung cancer; melanoma	
Ipilimumab	Anti‐CTLA‐4 antibody	NCT04655157 (phase 1/2); NCT04511013 (Phase 2)	Melanoma; renal cell carcinoma (RCC); colorectal cancer; hepatocellular carcinoma; non‐small cell lung cancer	
Nivolumab	anti‐PD1 antibody	NCT04655157 (phase 1/2); NCT04511013 (Phase 2)	PD1 expressing tumors	
Spartalizumab	anti‐PD1 antibody	NCT02607813 (Phase 1)	Non‐small cell lung cancer; ovarian cancer; melanoma; other solid tumors	
Cobicistat	CYP3A inhibitor	NCT05503797 (Phase 2)	Recurrent or progressive CNS tumors harboring BRAF fusions; recurrent high‐grade glioma (HGG) harboring BRAF V600E mutation	

Here, we review the recent breakthroughs in anti‐RAF treatments, such as small molecule inhibitors targeting all the RAF isoforms that would eventually break the paradox. In addition, we also discuss the dimer‐directed inhibitors and other innovative solutions, critically evaluating their efficacy in overcoming real therapeutic limits as well as their potential function in inhibiting oncogenic RAS.

## 2 RAF proteins

**Fig. 1:**
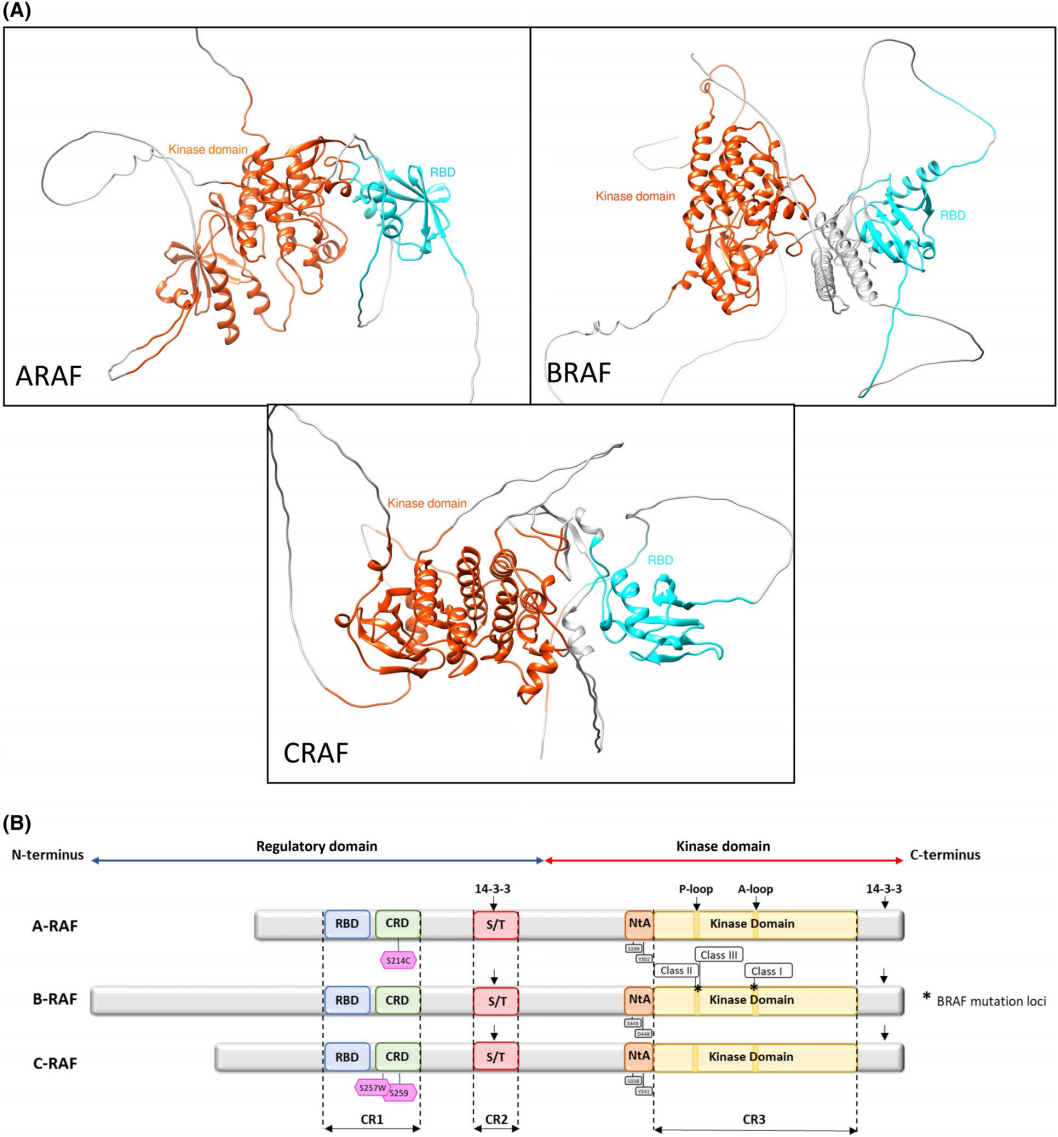
RAF isoforms and their predicted structures. (A) The kinase domain (orange) and the RBD (cyan) are conserved in each isoform. The detailed crystal structures are not available; predicted structures were obtained from the Alpha Fold database with the following identifiers. ARAF: AF‐P10398‐F1; BRAF: AF‐P15056‐F1; CRAF: AF‐P04049‐F1. Molecular graphics and the editing of the structures were performed with ucsf chimera (https://www.cgl.ucsf.edu/chimera), an extensible molecular modeling system developed by the Resource for Biocomputing, Visualization, and Informatics at the University of California, San Francisco, with support from NIH P41‐GM103311. (B) The three RAF proteins, A‐, B‐, and CRAF share three conserved domains; CR1, CR2 (regulatory domain), and CR3 (kinase domain). CR1 contains a RBD and a Cys‐rich region (CRD) and is essential for binding to RAS at the plasma membrane. CR2 includes a serine/threonine (S/T) phosphorylation site and a binding site for 14‐3‐3 protein. The kinase domain CR3 contains the phosphorylation loop (P‐loop), and the activation loop (A‐loop), and is responsible for RAF dimerization and MEK1/2 phosphorylation. At the C terminus of each RAF protein, there is a secondary 14‐3‐3 binding site which promotes dimerization. The N‐terminal acidic region (NtA) is located next to CR3 and interacts with helix αC causing the formation of the RAF dimerization interface. The noteworthy mutations are represented in the domains. Adapted from Degirmenci et al. [206].

There are three isoforms in the RAF family, A‐RAF, B‐RAF, and C‐RAF/RAF‐1, originating from three independent genes (Fig. 1A). Three conserved domains—two in the N‐terminal regulatory domain (CR1 and CR2) and one in the C‐terminal catalytic domain (CR3)—are present in all RAF proteins [21]. CR1 contains a Cysteine‐rich domain (CRD) [22], which associates with the plasma membrane through binding to phospholipids present in it [23] and farnesyl groups of RAS proteins [24]. Furthermore, CR1 involves an RAS‐binding domain (RBD), which is essential for binding to RAS [25]. The serine/threonine‐rich CR2 domain contains a conserved 14‐3‐3 binding motif [26]. CR3 domain serves as the kinase domain [21] and includes the catalytic DFG motif and the regulatory αC‐helix domain and is in control of binding and phosphorylating MEK1 or MEK2. A region near the N terminus of the kinase domain is known as the N‐terminal acidic (NtA) motif. For ARAF (S299, Y301, Y302) and CRAF (S338, S339, Y340, Y341) to dimerize, two NtA residues must be phosphorylated. Unlike these two isoforms, BRAF is constitutively phosphorylated at the serine residues (S445, S446) and has an aspartic acid residue instead of a tyrosine (D447, D448) to impart a constitutive negative charge. This region interacts with the αC‐helix, resulting in the formation of the RAF dimerization interface [27, 28]. When the RAF activation loop is phosphorylated, the RAF structure switches from DFG‐OUT to DFG‐IN and from the αC‐helix‐out to the αC‐helix‐in orientation, leading to an active protein conformation [29]. In addition, previous and more recent structural studies have shown that the CRD and the 14‐3‐3 binding motif are the key regulators of RAF autoinhibition (Fig. 1B) [30, 31, 32, 33, 34, 35].

### 2.1 RAF activating phosphorylation and dimerization

In quiescent cells, the RAF proteins are found as monomers in the cytoplasm [36], where they lack a subcellular localization motif and are, therefore, stable and inactive prior to pathway activation [16].

There are several mechanisms to keep the RAF monomers in such a stable, inactive state, including autoinhibition [37], phosphorylation of negative regulatory sites, and binding of inhibitory proteins [38, 39]. To be activated, the RAF proteins must overcome autoinhibition mediated by the amino‐terminal domain. One example of this process is the autoinhibitory interaction between the CR1 and the kinase domain, which should be released to enable the transition to the active state [16, 32]. All RAF proteins selectively interact with GTP‐bound RAS [40], even if RAF enzymatic activity is not directly stimulated by this contact, but rather by the relocation of cytosolic RAF to the plasma membrane, which is an essential step in RAF activation [41]. The proteins are phosphorylated at two conserved tyrosine residues (Tyr‐340/Tyr‐341 in CRAF, not present in BRAF) and a highly conserved serine (Ser‐299 in ARAF, Ser‐446 in BRAF, and Ser‐338 in CRAF) in the regulatory region (NtA) [42, 43, 44], which results in a conformational shift accompanied or induced by reorganization of 14‐3‐3 binding sites, which is considered a prerequisite for further RAF activation.

RAF dimerization represents another key event in their priming process: Dimer can be formed with the kinase suppressor of RAS (KSR) proteins, which function as scaffolds for ERK signaling and are controlled by the extracellular signals and MEK [45], along with any other RAF family member (homo‐ or heterodimerization) [46]. Data have shown that RAS‐mediated signaling is dominated by BRAF/CRAF heterodimers, which also have the highest catalytic activity [47, 48]. A key conformational change happens when the C‐helix and the activation segment (AS) in the N‐ and C‐lobes, respectively, move into an active form. This event allows alignment of the hydrophobic residues in the regulatory R‐spine (L358 for ARAF, L505 for BRAF, and L397 for CRAF), which is next to the conserved RKTR motif in the dimer interface (DIF), and causes dimerization of RAF kinase [49, 50].

Several processes must occur for the RAF to return to a pre‐activation state. One of the first processes leading to RAF inactivation is the dephosphorylation of sites previously activated by phosphorylation. The crucial S338 N‐region site of C‐RAF has been discovered to be dephosphorylated by protein phosphatase 5 (PP5) [51]. Holderfield et al. [52] found autoinhibitory sites in the ATP‐binding P‐loop of BRAF dimers, and a phosphoproteomic study of the BRAF V600E mutant revealed an autoinhibitory site in the AS (S614B‐RAF) [53]. Activated ERK phosphorylates the RAF on numerous S/TP sites in a negative feedback loop, which is another significant mechanism that reduces the activity of all RAF isoforms [38].

### 2.2 The importance of MRAS/SHOC2/PP1c in RAS and RAF signaling

Another mechanism that promotes full activation of the RAF–ERK pathway is the dephosphorylation of an inhibitory site on RAF kinases by the MRAS–SHOC2–PP1 complex [54]. MRAS is structurally similar to HRAS, NRAS, and KRAS and shares most regulatory and effector interactions [55]. MRAS binds directly to SHOC2, which was originally identified in *Caenorhabditis elegans* as a positive regulator of the RAS pathway [56]. Additionally, SHOC2 is necessary for the tumorigenic features of tumor‐derived cell lines with RAS mutations [54]. Later, the catalytic subunit PP1 (PP1c) is attached to the MRAS–SHOC2 complex, thereby, recruiting the entire complex to the plasma membrane, where it activates RAF by dephosphorylating CRAF kinase at S259 (S365 in BRAF and S214 in ARAF). Notably, dephosphorylation only occurs on RAF associated with RAS proteins in the membrane [57]. A detailed analysis of this complex's biochemical and structural components has shown its remarkable capacity to control RAF specificity, and provide approaches to inhibit it and target the RAS–ERK pathway [55, 58, 59].

The RASopathies, including Noonan syndrome, which are driven by mutations that increase signal transduction activity downstream of RAS, are better understood because of these novel structures. These structural studies and the evidence for their increased binding to MRAS explain the gain‐of‐function mutations impacting SHOC2 and PP1C identified in Noonan syndrome [60, 61]. Interestingly, RAF mutations have been detected in RASopathies, where BRAF mutations are mostly related to cardio‐facio‐cutaneous and CRAF mutations to Noonan syndrome [62, 63]. Significantly, the molecular features provided by these novel structures can contribute to the identification of disease‐causing mutations, improving our knowledge of the signaling mechanisms at work in both physiological and pathological contexts. Additionally, they uncover novel areas in the complex that might bind to inhibitors, opening up new possibilities for therapeutic approaches [58].

### 2.3 RAF's role in cancer

RAF is the second most frequently mutated element of the RAS–RAF–MEK–ERK pathway in cancers, after the RAS oncogene. BRAF mutations' higher incidence is registered in melanomas (40–50% of cases), and at a lower frequency in thyroid (10–70% depending on the histology), colorectal cancer (10% of cases), and NSCLC (3–5% of cases) [64].

Depending on the mode of activation and signaling of the kinase, the broad spectrum of BRAF mutations in human cancer has been divided into three subclasses. Class I mutations are the most common and depend on the residue V600, which increases BRAF kinase activity by ~500 times. The mutation replicates the structural changes that take place after dimerization; hence, BRAF V600 mutants can signal as a monomer in a RAS‐independent manner even if RAS is necessary for these mutants to form dimers [17, 64, 65, 66, 67]. Class II mutations fall either in the activation segment or in the P‐loop. These mutations present an intermediate to high kinase activity that activates the pathway more efficiently than the wild‐type (WT) BRAF but to a lower extent as compared to the class I mutations. To initiate the signaling cascade, these class II mutants form dimers in an RAS‐independent manner [64, 65, 67]. Class III mutations are found in the P‐loop, in the catalytic loop, and in the DFG motif, and they either confer a lower kinase activity than the WT BRAF or completely abolish it. These mutants can engage the pathway only after the formation of heterodimers together with CRAF or WT BRAF. This dimerization process is RAS dependent: Thus, class III mutations strongly rely on the upstream signaling. Hence, these mutations are rarely found alone; instead, they are typically co‐expressed with other mutations that activate RAS [65, 67, 68].

BRAF can also be constitutively activated after deletions occur in the proximity of the αC‐helix, which is kept in the active conformation, similar to class I mutations [69]. Furthermore, the protein fusions with many counterparts entail deletions at the N‐terminal CR1 auto‐inhibitory domain, which results in dimer formation comparable to class II mutants [70].

Mutations in the two other isoforms of RAF, ARAF, and CRAF are very rare. Imielinski et al. [71] found low‐frequency mutations affecting both the isoforms (ARAFS214C and CRAFS257W and CRAFS259F). Like BRAF, CRAF fusion proteins are found in low‐grade pediatric gliomas, prostate cancer, melanoma, and pancreatic cancer. The C‐terminal CRAF kinase domain connects to an N‐terminal fusion partner with a dimerization domain in these mutants, creating an aberrant protein that can dimerize in an RAS‐independent manner [72].

Interestingly, RAF has an isoform‐specific role in tumorigenesis in different non‐RAF‐driven cancers, particularly in those driven by RAS mutations. BRAF has been proven to be important for the phosphorylation of ERK in RAS‐driven skin cancer [73] but is nonessential for the development of KRAS mutant lung cancer. Due to its kinase‐independent functions, CRAF ablation prevents lung tumor development by KRAS mutants [74, 75, 76] and is indispensable for the initiation of RAS‐driven skin tumors [77]. However, it is not required for the formation of pancreatic ductal adenocarcinoma (PDACs) [78, 79] and exerts a tumor‐suppressive effect in hepatocellular carcinoma [80]. Lastly, it was discovered that in NRAS‐driven melanomas, BRAF is essential for the early stages of the illness's development and its activity cannot be replaced by CRAF; however, in the late stages of the disease, both proteins are needed [81].

CRAF was found to be upregulated also in hepatocellular carcinoma (HCC) patients, where silencing it with siRNA or miRNA decreased the progression of HCC cells [82, 83].

## 3 Current therapies' limitations

The history of RAF inhibitors begins with sorafenib (Table 1), which was initially intended to target RAS‐driven cancers but failed to achieve its purpose [84, 85]; it was also evaluated for the treatment of melanoma BRAFV600E patients, exhibiting only minor responses [86]. However, given the dramatic role of this mutation, efforts in the field contributed to the development of ATP‐competitive inhibitors with high specificity for monomeric (V600‐like) BRAF (from now on RAFis). Vemurafenib and dabrafenib were the first characterized and approved molecules for clinical use in melanoma [87, 88], followed by encorafenib (Table 1) [89].

**Fig. 2:**
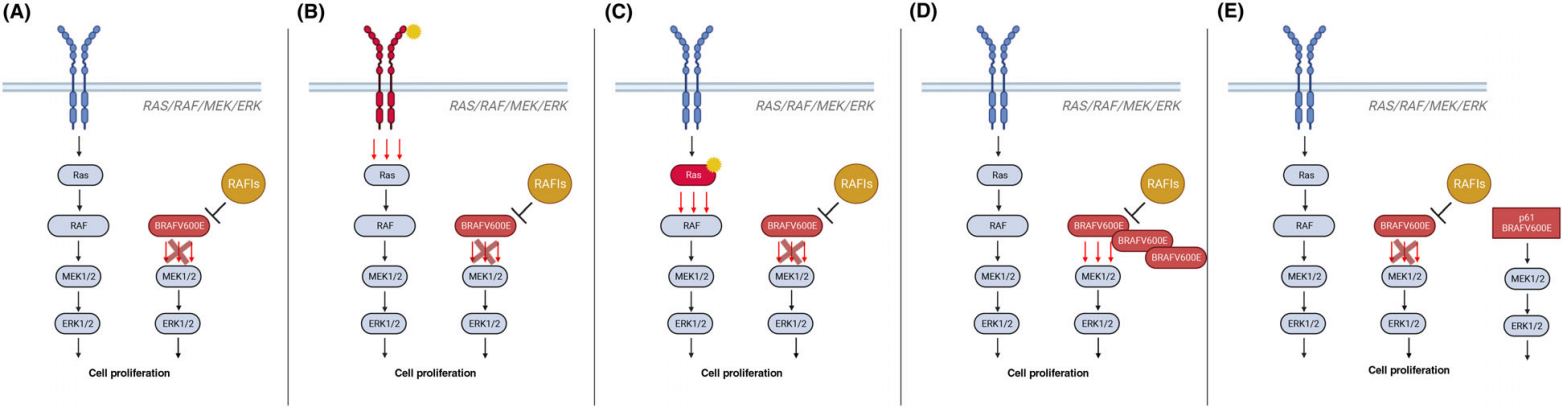
Mechanisms of resistance to RAFis. (A) The overactivation of the MAPK pathway is abolished in response to RAFi, which inhibits the growth of tumor cells. The reactivation of the MAPK pathway by both RTKR (B) and RAS (C) mutations, and subsequent disease relapse, mediate the loss of dependency on BRAF^V600E^ signaling. (D) A “sponge effect” occurs when there is an excess of mutant target due to BRAFV600E copy number gain. This indicates that while all drug molecules are bound, there are still free targets that are capable of initiating the signaling. (E) Splice variants that dimerize and prevent drug binding, like p61 BRAFV600E, reactivate the pathway, and cause resistance. Created with Biorender.com.

Melanoma patients treated with RAFis usually benefit from a good progression‐free survival (PFS) (around 6 months), but often relapse up to 1 year later [90, 91, 92]. The cases of resistance are usually associated with mutations in which ERK activation depends on RAF dimerization (presented in Fig. 2), RTKs and RAS protein alterations [93], BRAF copy number gains [94] splice variants (e.g., p61 BRAFV600E, which acts like a dimer) or fusions [17, 95], and MEK mutations [96].

Another concern is regarding the inefficacy of these compounds in contexts driven by RAF dimers, that is, BRAF mutations other than V600 (except for controversial rare cases) [97, 98, 99, 100], and tumors harboring RTK or RAS mutations [101]. Surprisingly, RAFis generate an opposite effect: They bind to WT RAF protomers priming their dimerization and then triggering the cascade, thereby, resulting in a process known as “paradoxical activation” of the pathway. In BRAFV600 patients, this peculiar event was correlated with the development of keratoacanthomas and squamous‐cell carcinomas, which are noninvasive and readily removed hyperplasia harboring RAS mutations [102, 103]. After approximately a decade of research, it was shown that this molecular process, activated by upstream signaling (RAS dependency), derives from the allosteric regulation mediated by both drug and RAS‐GTP binding to RAF protomers (both WT and BRAFV600 mutants) [104, 105, 106]. The contact with the drug promotes a series of events that include BRAF autoinhibition state release [68, 107], RAS–RAF interactions [106, 107], and homo‐ or heterodimerization of the protomers [17, 28, 67]. Additionally, this appeared to be inversely correlated with the dosage, as pERK signaling is triggered at low doses of inhibitor, but entirely quenched at higher concentrations [108], where the inhibitors occupy both protomers. Even if the precise mechanism is still unknown, it is evident that the paradoxical activation limits the use of RAFis in circumstances where RAS mutations may already be present or emerge as a resistance mechanism.

In conclusion, when triggered by upstream signals, RAFis can cause dimerization of WT‐RAF protomers. However, as RAFis are highly selective for monomers, they cannot prevent dimer formation accounting for their pathway activation (paradox). Resistant cases, RAS‐driven malignancies, and class II and III BRAF mutant tumors cannot be targeted by these compounds for the same reason. It is, thus, clear that identifying dimer‐directed treatments may improve therapy efficiency for RAS–RAF–MEK–ERK‐dependent malignancies.

### 3.1 Structural insights

Upon thorough structure/function investigation, an explanation for the peculiar and contrasting behavior of RAFis has been provided and is linked to their spatial disposition in the RAF catalytic domain. Therefore, an insight into the molecular basis of targeting a kinase is necessary to appreciate the molecular explanation for the issues linked to the paradoxical activation, and the strategies undertaken to overcome them.

**Fig. 3:**
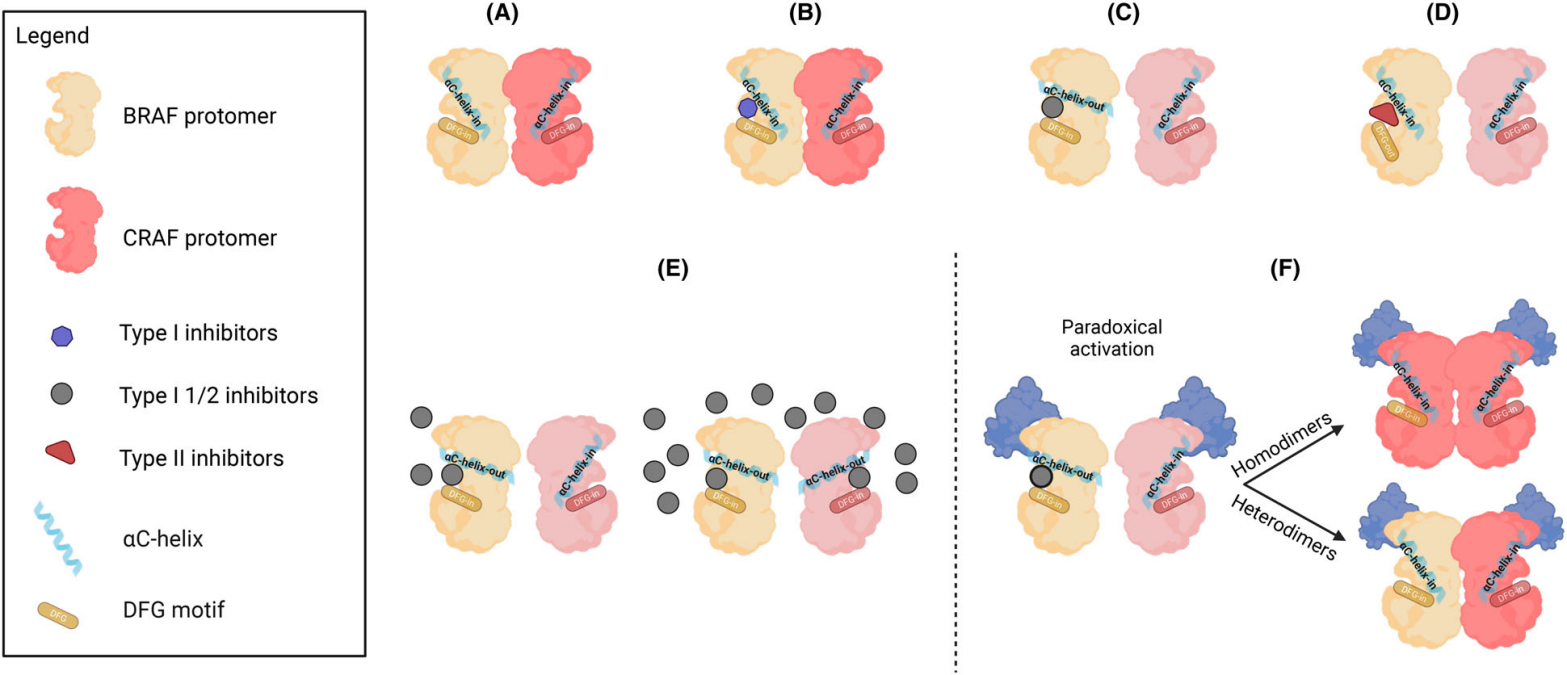
Examples of BRAF‐CRAF heterodimers formation in the presence of different kinase inhibitors starting from a condition of a drug‐free dimerization. (A) Type I, I ½, and II compounds are ATP competitors that bind to different conformations of the proteins, such as (B) αC‐IN/DFG‐IN (CIDI, for example, the GDC‐0879 BRAF inhibitor), (C) αC‐OUT/DFG‐IN conformation (CODI, e.g., BRAFis), (D) αC‐IN/DFG‐OUT (CIDO, e.g., sorafenib), respectively, represented in the binding pocket with different colors (see the legend). Starting from a physiological condition (A) (αC‐IN/DFG‐IN), BRAFis lock RAF kinases in an αC‐OUT/DFG‐IN state (C), and, at low concentrations decrease the affinity for the second protomer binding (negative allostery, E, left). At high concentrations of inhibitors, however, all the protomers are occupied and the negative allosteric effect is neutralized (E, right). Paradoxical activation: the same allosteric interaction triggers an opposite effect in a context driven by high RAS‐GTP levels and WT RAF: when bound to RAS‐GTP, the complex transactivates the second protomer of BRAF or CRAF, which adopts a configuration detrimental for drug binding but allows signal transmission and homo‐ or heterodimerization, ultimately activating the paradox (F). Created with Biorender.com.

In general, the kinases switch between active and inactive state depending on the spatial conformation of two different regions in the RAF ATP‐binding pocket, the αC‐helix and the DFG motifs, which can be in an “IN” or “OUT” orientation. Proteins are active when both regions are in the “IN” state, which allows dimerization [109]. Consequently, kinase inhibitors have been classified by their affinity for a certain configuration. For example, type I, I ½, and II compounds are ATP competitors that bind to a different conformation of the proteins, namely, (a) αC‐IN/DFG‐IN (CIDI, e.g., the GDC‐0879 BRAF inhibitor [107]), (b) αC‐OUT/DFG‐IN conformation (CODI, e.g., RAFis), (c) αC‐IN/DFG‐OUT (CIDO, e.g., sorafenib) [110, 111, 112]; the three different conformations compared to the physiological dimers are presented in Fig. 3A–D.

RAFis, thus, bind and lock RAF kinases in a αC‐OUT/DFG‐IN state. As shown in Fig. 3E (left), this conformation, at low concentrations of the inhibitor, decreases the affinity for the second protomer binding (negative allostery). Altogether, this explains the strong selectivity of type I ½ inhibitors for monomers rather than dimers, accounting for both the high therapeutic success in class I BRAF mutations, where single protomers are the driving oncoproteins and the inefficient block of dimers driving signaling (i.e., class II and III and RAF–MEK–ERK upstream mutations). However, in the latter setting, with WT RAF and high RAS‐GTP levels, the same allosteric interaction triggers an opposite effect (Fig. 3F) [105]. After binding to a CODI, the protomers are first released from their autoinhibited state [107], primed to RAS‐GTP binding due to allosteric [105] or spatial localization events [113], and thereby, transactivating a second protomer of BRAF or CRAF, which adopts a conformation that is detrimental for drug binding (αC‐IN), but that allows signal transmission and homo‐ or heterodimerization, ultimately activating the paradox [105]. As shown in Fig. 3E (right), at higher doses of inhibitors, all the protomers are occupied and the paradox is neutralized [108, 114]. Therefore, the research for novel RAF inhibitors has been focused for years on compounds that are able to block not only monomeric RAF (V600 mutations) but also WT BRAF and CRAF protomers to prevent dimerization.

## 4 Strategies to overcome the limitations

The first attempt to stop ERK reactivation in the aforementioned cases involved the use of targeted therapies against MEK (MEK inhibitors, MEKis), as it is the direct RAF effectors in the cascade. They have been approved both as a single therapy [115] and in combination with RAFis for melanoma [87, 116], NSCLC [117], and in 2022, the FDA granted the agnostic approval for the treatment of unresectable or metastatic solid BRAF V600E tumors, excluding colorectal cancers, that are intrinsically resistant to RAFis [118]. This approach, based on prevention of feedback activation [119], improved the treatment outcome and delayed resistance occurrence, especially when considering upstream (e.g., RAS mutations) or collateral (e.g., CRAF dependency) pathway reactivation [94], but did not completely address the problem [120, 121, 122]. Furthermore, there is a lack of clinical evidence to justify the combinatorial use for class II and III BRAF mutations [72], and it has not been approved for RAS mutant cancers [2]. Therefore, several compounds, such as pan‐RAF inhibitors and paradox breakers, were designed and developed to circumvent these difficulties.

### 4.1 Pan‐RAF

Considering the structural evidence and the idea of targeting both the dimer counterparts, pan‐RAF potent inhibitors (pan‐RAFis) of all the RAF isoforms were considered to be a promising option to prevent the limitations of type I ½ monomer‐selective drugs. A wide variety of compounds (αC‐helix‐IN/DFG‐OUT ‐type II) were generated with the objective of binding with the same affinity (no negative allostery) to the protomers of all the RAF proteins, preventing their transactivation, and retaining potency against RAF dimers (listed in [123]). Nonetheless, they still caused a mild grade of paradoxical activation [124, 125, 126, 127]. Indeed, as later explained by Karoulia et al. [105], locking the αC‐helix in the “IN” position can actually stabilize the second protomer in another “IN”‐activated state, favoring the dimerization and thus triggering the cascade. In addition, a promising preclinical efficacy for monotherapies in the clinics was not observed: Firstly, as these compounds can bind to WT RAF also in untransformed cells, pan‐RAFis were predicted to have a narrow therapeutic index [105]. This is the case of LY3009120 (Table 1), which, despite multiple encouraging experimental outcomes, could not achieve beneficial effects at the highest tolerable dose in the clinics (NCT02014116) [128]. Second, even if some trial responses are still awaited (NCT02607813, NCT04985604), the data available indicate that patients showed only minimal response to pan‐RAFis such as TAK‐580 and belvarafenib monotherapies [129, 130].

However, the benefits of these compounds have been proved in vertical combination with MEKi and ERKis in PDAC [94, 131, 132, 133]. This is further corroborated by the finding that certain drugs (such as MEKis) may increase RAF dimers or RAS‐GTP levels, which could augment the sensitivity of RAS mutant tumors to pan‐RAF inhibitors [134]. For example, the combination of belvarafenib (Table. 1) with cobimetinib (a MEKi, Table 1) showed potential effectiveness in NRAS mutant melanoma (NCT03284502) [135], while further studies are ongoing (NCT04835805).

Lifirafenib (BGB‐283) (Table 1) is another pan‐RAF inhibitor that also targets EGFR [136]. It demonstrated a favorable safety profile and efficacy against BRAFV600‐mutant solid tumors, including melanoma and KRAS‐mutant NSCLC (NCT02610361) [137]. Interestingly, when combined with MEKis, lifirafenib significantly blocked KRAS signaling in preclinical models [138]. This justified its recent translation to a phase Ib clinical study (NCT03905148‐rectruiting) in combination with mirdametinib (PD‐0325901, Table 1) among other MAPK alterations, for KRAS mutant NSCLCs patients.

Another interesting example is represented by LXH254 (Table 1), an inhibitor of BRAF and CRAF but not ARAF [139]. Unfortunately, the phase I study on LXH254 monotherapy (NCT02607813) for KRAS mutant patients did not show promising results, with only a minority of patients exhibiting disease stability [140]; this variability could be related to the higher or lower dependency of the tumors on ARAF, but, in general, ARAF expression is not low [141]. Despite its failure as a monotherapy, its combination with MEKis appeared promising in preclinical investigations [139], and with both MEKis and an ERKi (LTT462, Table 1) in clinics. For example, trials for the combination of LXH254 with LTT462 or trametinib (Table 1) are currently active but not recruiting NSCLC and melanoma patients (NCT02974725 and NCT04417621). In the former, a good safety profile and preliminary efficacy were observed in BRAF mutants [142], while in the latter, the combination with LTT462 or trametinib appears to be promising in NRAS‐mutant melanoma patients [143]. These findings provide new hope for RAS mutant patients; however, it is unclear whether these approaches can be applied to all scenarios where the signaling is dependent on dimerization (e.g., class II or III BRAF mutants). In addition, in regard to ARAF: LXH254 spares this isoform, and ARAF mutations were observed in cases of belvarafenib resistance (HM95573) [19]. Therefore, further research is necessary to evaluate its role in both responses to therapies and patients' relapse. For example, it could be interesting to investigate whether pan‐RAFis demonstrate optimal effects on malignancies that are more dependent on BRAF and CRAF [144]. Finally, in preclinical studies, KIN‐2787 (exarafenib) displayed good potency against all classes of BRAF mutants, as well as NRAS and KRAS mutants [145], but not against WT RAS/RAF; therefore, a dose escalation clinical trial is now underway (NCT04913285).

The most recent advances in the field categorized the pan‐RAFis into two classes: inhibitors binding with equivalent potency to monomers and dimers (mRAFis, e.g., lifirafenib, TAK580, LY3009120‐Table 1) and compounds selective for dimers (dRAFis, e.g., sorafenib, belvarafenib, LXH254), an effect probably induced by the latters' αC‐helix stabilization toward the “IN” conformation during RAF dimerization [144, 146]. The authors proposed a triple combination of a monomer selective (dabrafenib) + dimer selective (LHX254) + MEKi (trametinib, which would be targeting the RAF–MEK complex), which potently suppressed the pathway in PDXs (with lower toxicity than LHX254 + trametinib only) and in a patient with advanced CRC [146]. The idea of combining a type I ½ and a type II RAF inhibitor was also predicted by modeling and thermodynamic studies and verified in cellular models by Rukhlenko et al. Indeed, during the transactivation process, the drug's affinity for the second protomer is drastically reduced [108, 147] because the dimer structure is thermodynamically favored when composed by a drug bound and a drug‐free protomer [148]. This implies that the dimer could be successfully inhibited by molecules like type II inhibitors that would fit in the dimeric structure constituted of a free protomer coupled with an inhibited one [113, 149]. Even if the combination, dabrafenib + LHX254 + trametinib, has not reached the clinics yet, a similar vertical triplet treatment trial for the combination of dabrafenib, LXH254, and LTT462 (ERKi) is presently ongoing in advanced stage CRC patients (NCT04294160).

In conclusion, this classification defines RAF proteins as groups exhibiting different structural states and acknowledges that this spatial configuration diversity reduces the efficacy of monotherapies.

The combination of different types of RAF kinase inhibitors targeting diverse structural states is then a brilliant novel approach for overcoming both resistance and paradoxical activation. However, further research on selective dRAFis for combinatorial treatments is required, as the previous compounds were developed as pan‐RAFis and thus, retain some potency against monomers [150].

### 4.2 Paradox breakers

Given the well‐documented effects of the paradoxical activation, many independent investigations focused on molecules termed “Paradox‐Breakers” (PBs). As previously stated, pan‐RAFis were considered to be good PBs, but after initial enthusiasm, a minimal paradoxical activation was observed. Apart from pan‐RAFis, which have a good potential in combinatorial treatments, specific efforts in this field have culminated in the discovery and characterization of two pure PBs, PLX4072 and PLX8394 (Table 1) [151].

These compounds fall into the category of type I ½ inhibitors, similar to RAF inhibitors (RAFis). They operate by binding to the ATP binding pocket of the RAF protein, locking it into a CODI conformation. Notably, they possess the unique ability to stabilize the R506 residue in the OUT position within the αC‐helix. This altered conformation is less likely to facilitate interactions with RAS‐GTP compared to the IN conformation typically observed with RAF inhibitors [105].

PLX8394 inhibited the dimerization of BRAF homodimers and BRAF‐CRAF heterodimers, but no activity was observed on homo‐ or heterodimers of CRAF and ARAF. Thus, the molecule demonstrated high potency against class I and II BRAF mutants but showed only partial effectiveness against class III alterations or in the context of RAS mutations [152], which are more dependent on CRAF [153]. However, upon CRAF overexpression, the RAS–RAF–MEK–ERK pathway was activated [154], which is not surprising, because the molecule is BRAF‐specific, unable to efficiently prevent CRAF dimerization [152], and it has been reported that the paradox can be driven by CRAF bound to BRAF‐specific inhibitor in BRAF−/− cells [108, 147].

Regarding resistant tumors, BRAF fusions responded to PB treatment in specific genetic backgrounds [152, 155]: In some of these cases, paradoxical activation was eventually observed, most likely because protein fusions can alter the drug's allosteric interactions and transactivate the dimer partner. These last aspects justify a thorough examination of the genetic signature when projecting clinical translation. In this regard, a phase I/II trial (NCT02428712) is now underway to assess the clinical utility of a PLX8394 therapy with preliminary encouraging outcomes on BRAF‐mutant tumors [156]. Furthermore, a phase II trial of this drug in combination with cobicistat to improve its bioavailability has been initiated, but it is not yet recruiting patients with BRAF fusions and CNS BRAF class I and II mutations (NCT05503797).

Lately, a compound known as C1a (Table 1) was generated from a library of quinazolinic analogs [125] and demonstrated PLX8394‐like behavior, with good activity against BRAF mutant cell lines or PDX and no MEK–ERK activation in RAS mutant and WT BRAF as compared to RAFis [157]. Furthermore, it demonstrated efficacy against RAF‐dimer‐dependent resistance models [157], as well as the ability to cross the brain–blood barrier. Interestingly, since the existing therapies (both RAFis monotherapy and combination with MEKis) are poorly bioavailable in the brain [158, 159], this peculiarity, which is driven by a relatively low molecular weight (MW 461 Da), could play a significant role in the treatment of melanoma brain metastases. C1a effects were confirmed in cellular models and PDX that relapsed after the combination therapy, resulting in an overall tumor remission achieved at very low drug dosages [160]. Although clinical efficacy and resistance in different genetic scenarios have not been established, the properties of this compound appear promising and undoubtedly highlight the search for novel molecules that, while overcoming the paradox, would bring new hopes to relapsing patients and address the poorly investigated aspects of the disease. Finally, details of C1a localization into the binding pocket, currently unknown, could be considered for gaining structural insights into the unresolved mechanism of paradoxical activation.

### 4.3 Novel allosteric inhibitors

Type III and type IV allosteric inhibitors (namely, molecules that bind sites, respectively, close or secluded from the catalytic pocket [112]) were studied and developed to possibly lower the emergence of resistance counter‐mechanism by targeting a site other than the catalytic pocket [161].

#### 4.3.1 Type III

PHI1 (Table 1) is a type III RAF allosteric inhibitor particularly selective for αC‐helix “IN” conformation, and binding to a previously unknown allosteric site in this area. This dRAFis was able to bind a single protomer and induce positive cooperativity toward the second. This molecule demonstrated preclinical efficacy against dimers from class II and III BRAF mutants, and cases of resistance to RAFis, but no activity on RAS mutant cell lines [162]. This is a single preliminary study and deserves further investigation, which will open the way for the development of RAF inhibitors with a novel mechanism of action.

#### 4.3.2 Type IV–dimer interface inhibitors

With the idea of targeting the dynamic interactions between the RAF proteins, two structure‐guided investigations aimed at targeting the protein–protein interactions (PPIs) present at the DIF to inhibit the RAF node in RAS or dimer‐dependent BRAF mutant or resistant cancers. After a structure‐guided study, macrocyclic peptides with high affinity for the RAF's DIF were developed as type IV allosteric inhibitors and demonstrated good efficacy in preventing paradoxical activation induced by vemurafenib [163]. Gunderwala et al. used the same computational model to generate braftide (Table 1): This peptide effectively inhibited both a mutant and WT BRAF without causing paradoxical activation nor negative allostery; it also demonstrated activity toward BRAF fusions and showed an increased effect in combination with dabrafenib. Similar to proteolysis targeting chimeras (PROTACs), braftide segregation of BRAF in an inactive state prompted the RAF–MEK complex degradation in the proteasomes, although the mechanism has not been characterized [164]. These are promising but unique examples of compounds that are able to target the DIF as no small molecules have been developed for this purpose to date.

### 4.4 Proteolysis targeting chimeras

Targeted protein degradation is considered another valuable approach to overcome the limitations posed by the classical RAFis. With the objective of decreasing a target protein's levels by degradation, the PROTAC approach is a promising technology in this area: This system is based on small molecules that function as heterobifunctional degraders consisting of two ligands connected by a linker. One component recruits and binds to a protein of interest (POI), whereas the other recruits and binds to an E3 ubiquitin ligase. The POI and the ligase are, therefore, in close proximity so that the target can be ubiquitinated and then degraded by proteasomes. Given their ability to interact with a target, its commercially available inhibitors are utilized in PROTAC as warheads that bind the POI. In the recent years, numerous attempts have been undertaken to produce PROTACs that can specifically target oncogenic proteins such as BRD4, BRD9, ALK, and CDK4/6 [165, 166, 167, 168, 169, 170, 171, 172], and thus, several research groups tried to develop PROTACs that could degrade RAF proteins in order to bypass RAFis limitations.

Chen et al. were the first in the field to describe a PROTAC approach. They used rigosertib (RGS) as a warhead, a small anticancer drug designed to target PLK1 but that can also mimic RAS and interact with RBD. Since BRAF's mutations do not fall into the RBD, RGS is insensitive to its mutational state. Hence, it was used as a warhead that binds RAF and linked to pomalidomide (that can interact with cereblon (CRBN) E3 ligase). This molecule can degrade up to 80% of BRAF, but only at high concentrations, indicating a scarce potency [173].

Since 2020, several groups worked on the development of PROTACs directed versus BRAFV600E based on available RAFis vemurafenib, dabrafenib [174, 175], BI882370 [174, 176], and the PB PLX8394 [177]. The aforementioned compounds were linked to either a Von Hipple–Lindau (VHL) E3 ligase [175] or CRBN E3 ligase [174, 176, 177] binder to induce the degradation. All of them demonstrated advantages over the single molecules and were active at nanomolar concentrations [175, 177], with a fast kinetic degradation [174, 176] with *D*_Max_ (maximal percentage of degradation) ranging between 80% and 95% [175, 177].

The effect of all the compounds on the degradation of WT BRAF was tested and none of them decreased its levels. Alabi et al. [175] suggest that, although their PROTAC is able to bind the protein, the ternary complex (BRAF‐PROTAC‐E3) is characterized by weak interactions that do not allow the degradation of the target.

Another advantage of the PROTACs is the longer inhibition of the RAS–RAF–MEK–ERK pathway after the interruption of the treatment compared to the classical inhibitors [174, 175, 177]. A shorter inhibition of the pathway was only observed for the BI882370 PROTAC compared to its “parental inhibitor” [176]. However, the paradoxical effect was still triggered by the PROTACs based on vemurafenib, dabrafenib, and BI882370 when administered to WT BRAF cells and a high level of upstream activation, especially RAS activation [175, 176]. As expected, the effect was not observed with the PB PLX‐8394 [177], indicating that the paradoxical activation, which is related to how the small molecules interact with the target, cannot be overcome by adopting this strategy.

The efficacy of PROTACs might not be exclusive to the targeting of BRAF and its mutants. Drosten and Barbacid suggest that PROTACs targeting CRAF might have great success in the RAS‐mutant context, as they may recapitulate the effects obtained with the genetic ablation of this gene in GEM models, which are driven by the CRAF's kinase‐independent function [76, 178, 179]. This strategy, however, cannot be applied yet owing to the lack of molecules that can specifically bind to this isoform. Furthermore, as suggested by Poulikakos et al. [144], due to its role in resistance to RAF‐dimer inhibitors belvarafenib and LXH254, ARAF degradation by PROTACs could represent a future solution for overcoming resistance to this novel but promising pan‐RAFis.

### 4.5 Immunotherapies

In the recent years, immunotherapies have become increasingly important as first‐line treatments for various cancers, either as monotherapies or in combination with chemo‐ or targeted therapies [180, 181]. Thus, this strategy has also been exploited with the aim of improving the efficacy of RAF inhibitors. Most of the immunotherapies target immune checkpoints expressed by tumor cells that negatively regulate T‐cell activation, impairing cell‐mediated immune response, such as programmed cell death 1 (PD‐1) as well as cytotoxic T‐lymphocyte antigen 4 (CTLA‐4), expressed by T‐lymphocyte, and programmed cell death ligand 1 (PD‐L1).

Since 2010, numerous studies provided the biological rationale for the use of immune checkpoint inhibitors (ICIs) in cancers along with RAF mutations. Khalili et al. [182] reported an increase in the expression of IL‐1α and β cytokines enhancing T‐lymphocyte suppression by promoting PD‐L1 in melanocyte and melanoma cell lines. Furthermore, the treatment with RAFis increased the expression of PD‐L1 in resistant melanoma cells through the reactivation of the pathway [183]. Recently, high levels of CD8^+^ have been associated with pan‐RAFis + MEKis combination therapy, supporting the idea of a triple combination with anti‐PD1/PD‐L1 therapies [133]. Even the tumor microenvironment can be affected by RAFis alone or in combination with MEKi. For example, these treatments reduced vascular endothelial growth factor (VEGF) levels, which are responsible for blood vessel anomalies in tumor regions [183, 184], or increased melanocyte differentiation antigens (MDA) and promoted T‐lymphocyte specific recognition antigen [184, 185]. All these molecular alterations amplified the tumor microenvironment's lymphocyte recruitment. Additionally, the development of novel small molecules alongside the use of new ICIs is opening up the potential for novel therapeutic combinations: for example, a recent study on mice allografts of brain‐metastatic melanoma (poorly immunogenic) revealed that PB “C1a” therapy induced an immunological phenotype, sensitizing the tumor to anti‐PD‐1 therapy. In addition, while for the C1a monotherapy relapse was observed in four of 10 mice, no tumor relapse was observed for the combination C1a + anti‐PD‐1 [160]. Altogether, these preclinical results justified the design of potentially more effective combinatorial strategies.

In clinical settings, the combination or sequence of ICIs and RAFi/MEKi was mostly studied in the BRAF mutant melanomas. Even if the first combination of vemurafenib and ipilimumab (Table 1) for BRAFV600 melanoma patients induced significant hepatotoxicity after only a few weeks of treatment [186], different combinations of ICIs and RAFi were better tolerated, and thus, one of them was approved by FDA during the recent years. In particular, an anti‐PD‐1 and anti‐CTLA‐4 (nivolumab, Table 1, and ipilimumab, respectively) enhanced the overall survival (OS) of BRAFV600E and non‐BRAFV600E melanoma cancer patients as single treatments or in combination (phase III CheckMate 067 clinical trial) [187]. Now, this represents one of the first line of standard care options for patients with unresectable/metastatic BRAF mutant melanoma [188]. To date, three clinical trials investigating the combination of ICIs and BRAFi/MEKi have documented a controllable safety profile [189, 190, 191], with two of these trials demonstrating encouraging preliminary efficacy [189, 191]. Many other clinical trials are actively recruiting to investigate the positive effect of ICIs with RAFis and MEKis in melanoma BRAF mutant cancers. For example, the safety and efficacy of a double combination of encorafenib/binimetinib (MEKi, Table 1) with nivolumab and ipilimumab is being tested in patients with advanced BRAF mutant melanoma in a phase 1/2 (QUAD01, NCT04655157) and phase 2 (NCT04511013) clinical trials. Sequential treatments are an important issue that clinicians face in their everyday practice, especially when different drug regimens show efficacy both in first‐ and further‐line therapies. Optimizing sequential treatments can improve treatment duration, efficacy, and minimize adverse events (AE) of ICIs and RAF target therapies. Some clinical trials such as SECOMBIT (NCT02631447) and DREAMSeq (NCT02224781) have demonstrated the efficacy of ICI treatment followed by targeted therapies; both of them observed an increase in the 2‐year OS (73% and 71%) for groups treated with ICIs in combination before receiving RAFis, compared to the ICI treatment after RAFis (65% and 51.5%) [192, 193].

Conversely, ICIs or their combinations with RAFis and MEKis have been tested, such as the “classic” oncogene‐addicted NSCLC, where the efficacy of immunotherapy is otherwise poor [194, 195]. However, it is known that not all the molecular subgroups of NSCLCs are the same; in fact, Guisier et al. [196] conducted a retrospective analysis on a heterogeneous group of NSCLCs, containing BRAF, HER2, MET, and RET as driver mutations, treated with ICIs and found that the response rate is similar to that of NSCLC patients without actionable mutations. Another study considered only BRAF V600E and non‐V600E mutant NSCLC patients treated with ICIs and reported a similar response rate and PFS in both groups [197]. Unfortunately, due to the small proportion of BRAF‐mutanr NSCLC, definitive conclusions about which sequential or combinational strategies are the best therapeutic choice are not definitive. While new combinational strategies are being tested, it is now widely accepted that immunotherapy represents one of the standard care options for non‐oncogene‐addicted metastatic NSCLC [198, 199]. Finally, a recently completed phase I clinical trial analyzed the efficacy and safety of a new pan‐RAF compound, LXH254, and an anti‐PD1, spartalizumab (PDR‐001, Table 1), against different solid tumors including NSCLC; however, the results of this study are not yet available (NCT02607813).

Another interesting therapeutic approach is the Adoptive Cell Transfer (ACT) treatments. Here, immune cells from patients or donors are firstly expanded and enhanced *in vitro* to be tumor‐specific, and then intravenously infused into the patients. Among them, the chimeric antigen receptor (CAR) T‐cell therapy is a novel approach that is used when ICIs therapies fail in patients [200]. In this case, the patient's T‐cell receptors are genetically modified to identify a particular tumor antigen and expanded before being reinfused into the patient. This therapy was successfully used against liquid cancers and in the recent years was applied to solid malignancies too. In particular, a recent *in vitro* study on BRAF mutant melanoma suggests that the combination dabrafenib‐trametinib interferes less than vemurafenib‐cobimetinib with the CAR‐T therapeutical efficacy, thanks to its lower inhibitory effect on the CD4^+^ and CD8^+^ T‐lymphocyte [185, 201]. Another example is the ACT tumor‐infiltrating lymphocyte (TIL) immune cells; they are isolated from excised tumors, then selected for those that are able to recognize the tumor‐associated antigens, expanded, and then reinfused into patients. This therapy is particularly effective in advanced melanoma patients who have failed ICI therapy and appears to be more effective when combined with BRAF and MEK inhibitors, which boost tumor immunogenicity [202].

Nowadays immunotherapies are efficient against a selected group of malignancies; however, new strategies have been developed in the past few years to improve the efficacy of immunotherapeutic drugs such that it can be combined with RAFis. For example, PF‐04136309, a small molecule that suppresses the CCL2‐CCR2 axis involved in immunosuppressive tumor microenvironment maintenance, thereby, increasing CD4^+^ and CD8^+^ activity and showing promising results [203]. Furthermore, with the discovery of new targets, novel targeted therapies will be developed, and, therefore, new combinatorial treatments will be designed, making immunotherapy a standard care option to improve therapeutic outcomes.

## 5 Conclusions

While RAFis provide considerable improvements in cancer treatment, they also exhibit some limitations, including drug resistance, paradoxical activation, and low efficacy for class II and III BRAF mutations and RAS‐driven malignancies. To circumvent such constraints, various strategies associated with type II inhibitor monotherapy approaches are being investigated, with a few displaying encouraging results. However, the most impressive outcomes derive from the combinatorial use of different small molecule inhibitors, and with immunotherapies. In the latter case, encouraging preclinical results on the combination of novel RAFis and ICIs warrants further research in this direction to detect potential biological vulnerabilities, and that, in general, immunotherapies can act synergistically with these inhibitors, to improve and prolong their efficacy. However, if the combinatorial treatments seem promising and feasible, the efficacy and safety of the proposed strategies are still under investigation in clinics (dose escalation trials). Notably, these innovative approaches have been proposed not only for RAFis‐resistant tumors but also on RAS‐driven malignancies. Indeed, while for a long time attempts to target RAS utilizing its downstream effectors were unsuccessful, in the recent years, novel findings justified the rationale of using RAF inhibitors in RAS mutant tumors as well [204]. Hence, the combination with the recently approved direct KRAS inhibitors needs to be further evaluated.

Conversely, new technological strategies such as the PROTACs provide novel design of RAF inhibitors, together with the new targetable features and novel type III and IV allosteric inhibitors. The recent advances in understanding cancer biology will also pave the way for the discovery of new targets: For example, the structures of autoinhibited and active BRAF‐MEK1‐14‐3‐3 complexes were recently reported, showing the underlying molecular mechanisms governing RAF modulation. These outcomes will provide unique options to treat RAF‐related malignancies and shed light on how 14‐3‐3 affects the paradoxical activation by RAFis, and hence, the susceptibility to these molecules [33, 205].

In addition, a recent analysis of the complex, MRAS‐SHOC2‐PP1, and its biochemical and structural components have revealed its remarkable capacity to control specificity for RAF and provide approaches to inhibit it to target the RAS–ERK pathway [55, 58, 59]. In conclusion, our molecular understanding of the signaling mechanisms in both physiological and pathological contexts and unexplored areas in the complexes that might be able to bind inhibitors, open a new realm of therapeutic strategies.

## Conflict of interest

The authors declare no conflict of interest.

## Author contributions

RS and CA conceived the study; RS, EB, PS, SVM, EG analyzed the sources and generated the figures; RS, EB, PS, SVM, EG, SN, DS, CA wrote and/or revised the manuscript.
